# Behavioral Plasticity in Response to Environmental Manipulation among Zebrafish (*Danio rerio*) Populations

**DOI:** 10.1371/journal.pone.0125097

**Published:** 2015-04-30

**Authors:** Anuradha Bhat, Melissa M. Greulich, Emília P. Martins

**Affiliations:** 1 Department of Biology and Center for the Integrative Study of Animal Behavior, Indiana University, Bloomington, Indiana, United States of America; 2 Department of Biological Sciences, Indian Institute of Science Education and Research-Kolkata, Mohanpur, India; Queen Mary University of London, UNITED KINGDOM

## Abstract

Plastic responses can have adaptive significance for organisms occurring in unpredictable environments, migratory species and organisms occupying novel environments. Zebrafish (*Danio rerio*) occur in a wide range of habitats and environments that fluctuate frequently across seasons and habitats. We expect wild populations of fish to be behaviorally more flexible than fish reared in conventional laboratory and hatchery environments. We measured three behavioral traits among 2 wild (U and PN) and 1 laboratory bred (SH) zebrafish populations in four environments differing in water flow and vegetation regimes. We found that the degree of plasticity varied with the type of behavior and also among populations. In general, vegetation increased aggression and water flow decreased latency to feed after a disturbance, but the patterns were population dependent. For example, while wild U fish fed more readily after a disturbance in vegetated and/or flowing habitats, fish from the wild PN population and lab-reared SH strain showed little variation in foraging across different environmental conditions. Zebrafish from all the three populations were more aggressive when tested in an arena with vegetation. In contrast, while there was an inter- population difference in shoaling distances, variation in shoaling distance across environmental conditions within populations was not significant. These results suggest that both foraging and aggression in zebrafish are more plastic and influenced by immediate context than is shoaling distance, which may have a stronger genetic basis. Our findings point to different underlying mechanisms influencing the expression of these traits and warrants further investigations.

## Introduction

Phenotypic plasticity is the ability of an organism with a given genotype to change its phenotype in response to changes in the environment. The ability of individuals, populations or species to switch between behaviors across situations can have important ecological and evolutionary implications. For example, phenotypic plasticity can play a role in the process of diversification and species range-expansion [1]. Several species show behavioral variation as an adaptive response to different environments [2, 3] and as an important strategy for coping with environmental variability [4, 5]. Behavioral divergence between populations can take place over shorter periods than in higher order clades [6]. Different ecological environments can impose strong divergent selection leading to radiation in behavioral responses among populations [7]. As long as there is minimal gene flow between populations, these radiations can drive evolutionary divergences. Here we study the behavior of zebrafish populations from different (lab-bred, lake, and pond) habitats and rearing conditions in a series of environments and measure the extent of behavioral variability within populations.

The ability to switch behavior is also related to early experience—juvenile cod from wild habitats that are more heterogeneous differed in their shoaling responses when moved between different habitats, whereas those from hatchery environments (plain habitats) responded in the same way across testing conditions [8]. The effect of habitat complexity on behavioral plasticity would depend on the early rearing experience [9, 10]. Learning and memory can also influence animals to adjust their behavior in variable environments. For example, although early research showed a genetic basis for antipredatory response in fishes, recent evidence from several species suggests that learning plays an important role in development of this behavior [11, 12]. Further, it has been demonstrated recently in populations of three-spined sticklebacks that learning and memory are influenced by habitat stability and predation pressure [13]. Members of a shoal observe the behavior of their shoalmates and are able to respond to predator threats more efficiently (through improved predator avoidance and escape responses) [14, 15, 16]. Additionally, shoaling with knowledgeable conspecifics can also improve foraging efficiency through increased detection of food resources [17] and attraction to novel food items [18]. Recent tests on Atlantic salmon have shown that combination of enriched environments with live prey provided to hatchery reared fish prior to release into the wild increased their post release survival rates [19]. Training hatchery reared fish through social learning, that is, learning by observing or interaction with shoalmates has thus been suggested as a useful tool for successfully restocking populations [20].

Pond/lake and river habitats are different in many respects, especially with regards to water flow, and could differ in water clarity, presence of vegetation and predators. These factors could be critical to fish populations for foraging, responding to predatory threats or even mating tactics. Indeed, stickleback populations exposed to different environmental conditions have been found to differ in the types of information they use to solve spatial tasks—fish from unstable river habitats rely less on visual cues than fish inhabiting visually stable habitats such as ponds [21]. Zebrafish, native to south and south-east Asia, occur across a range of flow and vegetation regimes (still-water lakes with thick vegetation to flowing clear water streams) [22]. Their natural environments often fluctuate in water flow and vegetation conditions across the year- habitat stability may vary between microhabitats especially during the dry seasons when certain regions get cut off from the main channels and these habitats might return to steady flowing conditions during the wet season. Hence individuals that can switch behavior in response to these fluctuations may have a survival advantage. Fish that have been reared in hatchery environments experience minimal heterogeneity in habitat or environmental variation. Here, we predict that given this variability in natural environmental conditions, one would expect zebrafish populations that evolved in the wild to show greater behavioral flexibility than fish evolving in a stable laboratory environment. Wild populations from different flow and vegetation regimes (e.g. lakes and streams) may also vary in degree of behavioral plasticity. For example, fish from stagnant lakes may exhibit similar plasticity to laboratory-bred populations than do fish from streams. Furthermore, some behavior patterns (e.g. foraging) can be strongly influenced by environmental cues while others are more constrained by correlated behavior. Here we ask the following questions with regards to feeding latency, aggression and shoaling behavior in zebrafish populations- 1) What are the effects of water flow and vegetation on behavioral response among populations from lab and wild rearing environments 2) Do populations differ in their responses when tested in tanks with/without flow and vegetation and lastly, 3) do zebrafish populations from different rearing environments (lake, stream and lab conditions) show variable plastic response when presented with fluctuating flow and vegetation conditions? Specifically, we tested our hypothesis that wild zebrafish would show greater variability in behavioral response than test subjects from lab bred populations when subjected to differing flow and vegetation regimes (i.e., with/without flow and/or vegetation).

## Material and Methods

### Model system

*Danio rerio* is a small cyprinid fish (~ 30mm SL), native to part of the Southeast Asian region- eastern and south western India [22, 23], Bangladesh [24] and Myanmar [25]. At the microhabitat level, these habitats differ in water flow, vegetation and turbidity [24, 26]. They swim in shoals of 2–10 individuals [27], typically in slow-moving streams and stagnant water bodies, paddy fields, low-lying floodplain lakes, ponds and irrigation channels [28, 29].

We used three populations of zebrafish for this study. ‘SH’ or Scientific Hatcheries, was developed in the early 1990s, and is available commercially from Scientific Hatcheries in Huntington Beach, CA. SH have evolved now for dozens of generations in stable, high density conditions similar to conditions in commercial hatcheries. We collected two wild populations in January- February 2007 from West Bengal state (in northeastern India). We collected ‘PN’ fish from an oxbow lake (approx. 180m wide and 10m deep in the middle of the lake) (located in North 24 Pargana district of West Bengal, India) with still water and floating and submerged vegetation. Zebrafish prefer shallower parts of the lake (within 1.5–2m deep) along the lake fringes. The lake fringes are also high in submerged (reeds) and floating vegetation (*Eicchornia crassipes*). Individual fish in this lake would have the opportunity to move readily from the deeper and less vegetated center of the lake to the shallower and densely vegetated fringes. The PN lake population is subjected to predatory pressures from birds (herons, kingfishers and cormorants) as well as large piscivorous fish (*Channa* spp., *Xenontodon cancila*, *Oreochromis mossambicus* ([22], pers. obs.). The other fish species that we collected from this habitat and share habitat space with zebrafish include *Esomus danricus*, *Colisa lalia* and *Aplocheilus panchax*. The ‘U’ population occurs in irrigation canals (along paddy fields in South 24 Pargana district of West Bengal, India), with slow moving water and floating/submerged vegetation, in a more uniform habitat. The channel is 15–20 m wide, and 0.5 m deep. This is a slow flowing habitat (water flow <1 m/s) consisting of dense vegetation (mostly, smaller macrophytes, and floating bryophytes *Lemna* spp.). Due to the smaller size of this habitat, zebrafish are subjected to lower predatory pressure from large piscivorous fish. Some predatory birds (kingfishers), however, are likely to be present. The substrate in both the lake and slow-moving channel habitat was predominated by silt and sand. Very similar vegetation and faunal records have been made in recent zebrafish studies in lake and stream habitats around this region (Bengal, Bangladesh, and Meghalaya) by [22], [24].

In the lab, we housed fish from all three populations in standard 19-l glass aquaria (40.5 X 21.5 X 26.5 cm^3^) with 15–20 individuals, under uniform conditions of a daily diet of fish flakes and brine shrimp, a 12:12 day-night cycle, and water temperature of ~26°C. Wild collected fish were kept in these conditions in the laboratory for 2.5 months before they were used for behavioural tests. This was done to ensure that all individuals used in the experiments were not stressed from the relocation.

### Behavioral Protocol

We tested fish in four large test arenas (106-l plastic translucent tanks, 45.2 x 84.8 x 45.5 cm^3^), each containing a different environmental treatment of vegetation and flow regimes (1- clear with flowing water; 2- vegetated with still water; 3-, clear with still water and; 4- vegetated with flowing water). We used wet rotor pumps to generate a water velocity of 14 cm/sec in two of the test arenas, and evenly distributed 4–5 submerged plastic plants to create a vegetated habitat in two of the test arenas. To avoid bias due to satiation, test fish were fed a day before the experiments were conducted. Sex ratio among wild populations in zebrafish has been observed to be 1:1 (Spence et al., 2007, 2008). We therefore created shoals of 6 (adult, 1:1 male-female ratio) individuals from a single population (U, PN, or SH), and introduced the shoal into one of the 4 treatment tanks (chosen at random). We allowed the shoal to acclimate for 20–25 minutes, and then measured 1) *Latency to Feed* (time for any fish to approach dry flake food offered at one end of the test arena), 2) *Aggression* (total number of chases initiated by any member of the shoal in 5 min), and 3) *Shoal Distance* (average of 10 estimates of the maximum distance between fish taken during a 5-min period). The ‘Latency to Feed’ was used as a measure of the inclination of fish to feed versus the extent of their wariness. The ‘Shoal Distance’ provided an estimate of shoal cohesiveness- larger the average distance between members, less is the cohesion. We then transferred the entire shoal into a second test arena with different environmental features (chosen at random without replacement), allowed them to acclimate for 20–25 minutes, and repeated the behavioral measures. We repeated this entire procedure until we had measured each shoal in each of the 4 test arenas. All experiments were conducted between 10.00–14.00 hours and the tanks were lit overhead with fluorescent lamps placed uniformly above all tank treatments. We tested a total of 16 unique shoals (96 individual fish) from the U population and 12 shoals each from PN and SH populations (72 fish from each). All behavioral observations were video- recorded using a digital camera placed directly overhead. Measurements of “latency to feed” were done directly while “Aggression” and “Shoal distance” were measured from the video recordings by two independent observers. Readings were then compared to check against any observer biases.

Zebrafish populations are widely distributed in this region and the species is designated as “least concern” by IUCN’s redlist of threatened species. Further, as the collections were made outside a reserved forest or protected area, we did not require prior permits or approval for collection in India. The field study did not involve any endangered or protected species. The protocol for this study was approved by the Institutional Animal Care and Use Committee of Indiana University (Protocol#: 07–074).

### Statistical Analyses

We performed a multivariate ANOVA (MANOVA) to study the effects of population, and tank-treatment type and their interactions effects on behavioral responses. Here, the dependent measures were the three behavioral responses (Latency to Feed, Aggression and Shoal distance), the 4 types of test arenas (with different flow and vegetation regimes) were the within-subject factors, while the 3 populations (U, PN and SH) were the between-subject factors. In order to test the effect of tank-treatment type on behavioral responses within populations, we then used repeated-measures (within groups) ANOVAs for the effect of tank treatment type (within subject factor) across each population. The assumption of sphericity was tested by the Mauchly’s test and wherever it showed a violation, a corrected value (Greenhouse-Geisser correction) of F was used. We further performed posthoc tests (with Bonferroni adjustments for multiple comparisons) to examine differences across pairs of treatment types within populations. Data on measures of Aggression (as these are count measures) was square-root transformed while measures of Latency to feed and Shoal distance were natural-log transformed to obtain normal and homoscedastic residuals. All analyses were conducted using SPSS version 16 [30].

## Results

### Effects of tank-treatment type and population on behavioral responses

The Mauchly’s test for the assumption of sphericity was met for all three behavioral traits (Aggression: χ^2^ (5) = 10.37, p = 0.065; Latency to feed: χ^2^ (5) = 6.94, p = 0.22; Shoal Distance: χ^2^ (5) = 9.55, p = 0.089). The results of the repeated measures MANOVA show that there was a significant overall within-subject effect of tank-treatment type (Pillai’s Trace = 0.746, F (9,306) = 11.252, p<0.001) as well as interaction effect for tank and population type (Pillai’s trace = 0.37, F (18,306) = 2.39, p = 0.001).

### Population differences in behavior

**Fig 1:**
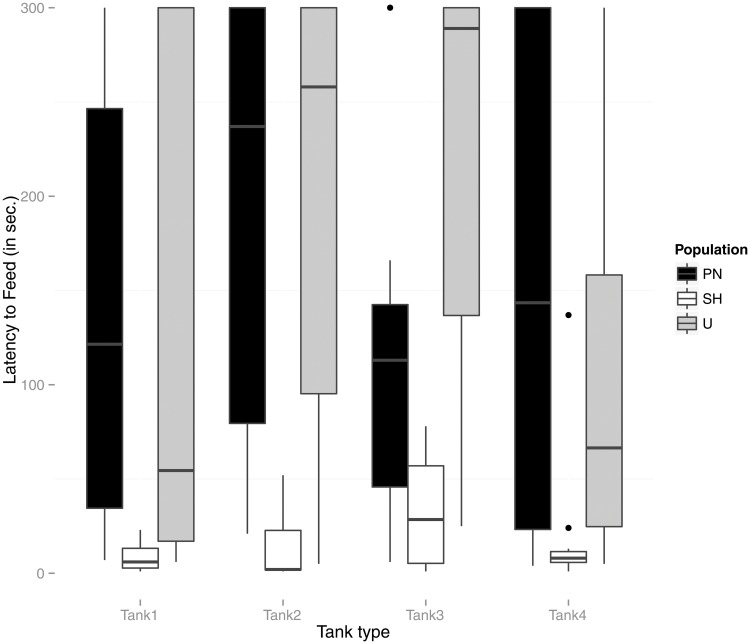
Box-plot representing population-wise measurements of Latency to feed (in seconds) in each tank treatment type (tank 1, tank 2, tank 3, and tank 4). Tank 1 (Flow, no vegetation), 2 (No Flow, vegetation), 3 (No flow, no vegetation) and 4 (Flow, Vegetation). Outliers are shown as solid dots.

**Fig 2:**
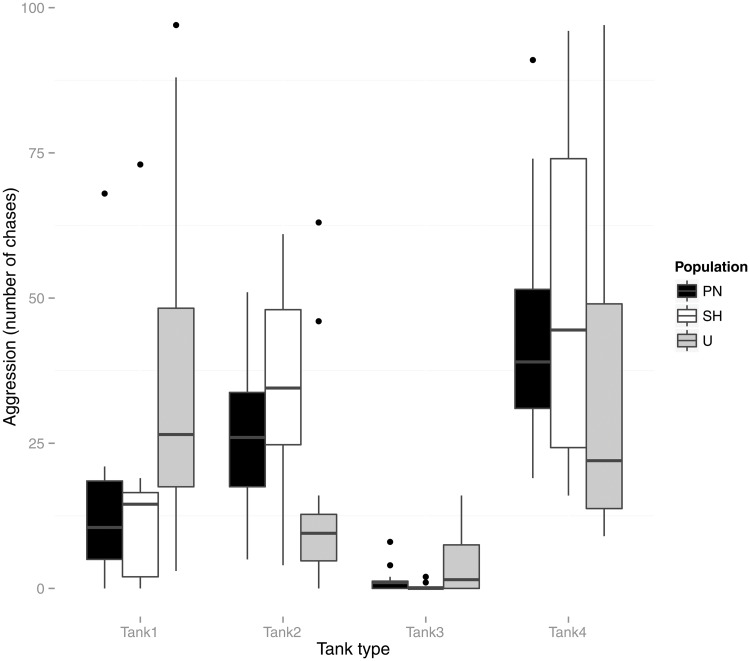
Box-plot representing population-wise measurements of Aggression (total number of chases initiated by individuals in a trial) in each tank treatment type (tank 1, tank 2, tank 3, and tank 4). Tank 1 (Flow, no vegetation), 2 (No Flow, vegetation), 3 (No flow, no vegetation) and 4 (Flow, Vegetation). Outliers are shown as solid dots.

**Fig 3:**
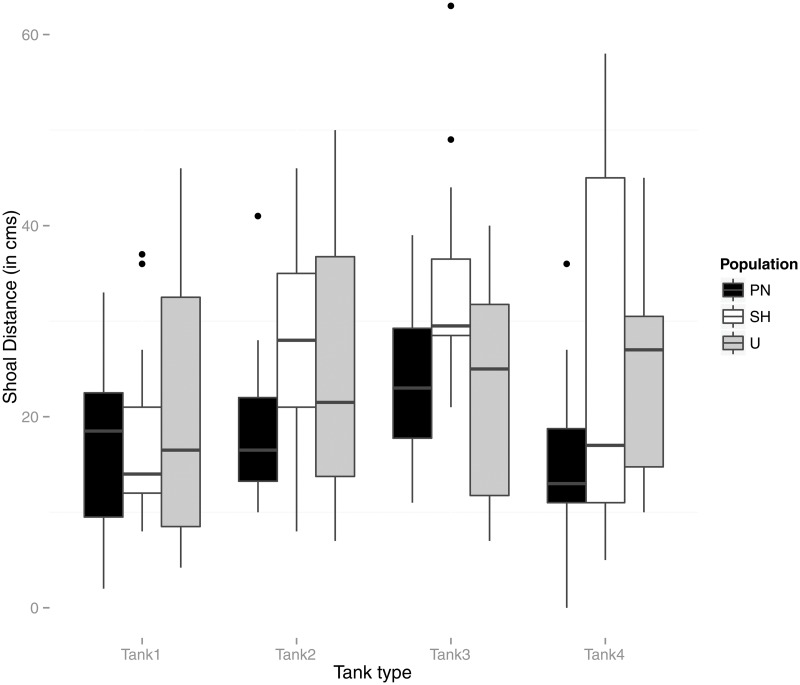
Box-plot representing population-wise measurements of Shoal distance (i.e. average distance of pairs of individuals in each shoal in cms.) in each tank treatment type (tank 1, tank 2, tank 3, and tank 4). Tank 1 (Flow, no vegetation), 2 (No Flow, vegetation), 3 (No flow, no vegetation) and 4 (Flow, Vegetation). Outliers are shown as solid dots.

Repeated measures ANOVAs on each behavioral trait response with tank treatment as within subject and population as between subject factor were conducted (Table 1, Figs 1, 2 and 3). No significant effect of either the tank treatment or the interaction (tank treatment X population) was found for Latency to feed measurements. Post-hoc paired comparisons for differences between populations revealed significant difference between both the wild populations (U and PN) with the lab (SH) population (SH Vs U; Mean difference = 2.47, p<0.001 and SH vs PN; Mean difference = 2.40, p<0.001). While tank treatment was found to significantly affect Shoal distance, interaction effects between tank treatment and population were not significant. Post hoc paired comparisons between populations revealed no significant differences. Tests for effects of tank treatment and population on Aggression showed significant main effect of tank treatment and interaction effects (tank treatment X population) (Table 1). Posthoc paired tests to compare pairs of populations, however, did not reveal significant differences.

**Table 1:** Repeated measures ANOVA on effects of population (between subject) and tank treatment type (within subject) on behavioural responses.

Behaviour	Factors	F(df)	P
**Latency to feed**	Tank treatment type	2.72 (3, 111)	0.05
	Tank treatment type X Population	2.27 (6,111)	0.04
	Population	47.4 (2, 37)	<0.001
**Aggression**	Tank treatment type	54.26 (3, 111)	<0.001
	Tank treatment type X Population	5.37 (6,111)	<0.001
	Population	0.003 (2, 37)	0.99
**Shoaling distance**	Tank treatment type	6.15 (3, 102)	0.001
	Tank treatment type X Population	0.89 (6,102)	0.50
	Population	2.30 (2, 34)	0.11

### Behavioral plasticity across environmental situations

Repeated measures ANOVA were performed within each population separately to test the effect of tank treatment for each behavioral response, followed by multiple paired post-hoc comparisons (with Bonferroni adjustments) between tank-treatments within populations. Populations were found to differ in the extent of behavioral plasticity to varying tank conditions (Table 2).

**Table 2:** Repeated measures ANOVA results.

Behaviour	Test value (df)	U	PN	SH
**Latency to feed**	F (df)	3.48 (3,45)	1.20 (2,22.6)	3.06 (3,33)
	p	0.02	0.32	0.04
**Aggression**	F (df)	14.15 (3,45)	30.09 (1.8,19.7)	24.21 (2,22)
	p	0.00	0.00	0.00
**Shoaling distance**	F (df)	1.04 (3,45)	3.10 (3,33)	4.79 (1.6,17.6)
	p	0.38	0.04	0.03

Within populations effects of tank treatment type on behaviour. Greenhouse-geisser corrected F values are shown wherever the data violated the sphericity assumption.

None of the three populations showed significant variation in feeding latency across tank treatments (p<0.01). While Shoal distance measurements among the wild U, and PN populations did not show significant plasticity across treatments (Table 2), there was a weak effect of tank treatment in the lab SH populations (p = 0.03). Pairwise comparisons (post-hoc tests) showed no significant differences among tanks except for one paired test (within the lab SH population) between Shoal distance in the tank with flow, no vegetation and the tank with no flow or vegetation (paired samples t-tests with p values adjusted by the bonferroni adjustment) (Tables 3, 4 and 5).

**Table 3:** Paired comparisons of mean differences across tank treatment types (1, 2, 3 and 4) for (log transformed) Latency to feed, (square-root transformed) Aggression and (log transformed) Shoaling distance measurements.

Tank Type	Behaviour	1: Flow, No Veg	2: No Flow, Veg	3: No flow, No veg	4: Veg, Flow
**1**	**Latency to Feed**		0.86	1.43	0.007
	**Aggression**		**2.38**	**3.98**	0.04
	**Shoaling Distance**		0.20	0.33	0.40
**2**	**Latency to Feed**			0.28	0.86
	**Aggression**			1.60	2.34
	**Shoaling Distance**			0.13	0.20
**3**	**Latency to Feed**				1.14
	**Aggression**				**3.94**
	**Shoaling Distance**				0.06
**4**	**Latency to Feed**				
	**Aggression**				
	**Shoaling Distance**				

Tank 1 (Flow, no vegetation), 2 (No Flow, vegetation), 3 (No flow, no vegetation) and 4 (Flow, Vegetation) for U population. Significant differences (after bonferroni corrections for multiple comparisons) with p<0.01 are shown in bold.

**Table 4:** Paired comparisons of mean differences across tank treatment types (1, 2, 3 and 4) for (log transformed) Latency to feed, (square-root transformed) Aggression and (log transformed) Shoaling distance measurements.

Tank Type	Behaviour	1: Flow, No Veg	2: No Flow, Veg	3: No flow, No veg	4: Veg, Flow
**1**	**Latency to Feed**		0.65	0.13	0.09
	**Aggression**		1.57	**2.52**	**3.10**
	**Shoaling Distance**		0.32	0.64	0.07
**2**	**Latency to Feed**			0.78	0.75
	**Aggression**			**4.08**	1.53
	**Shoaling Distance**			0.32	0.25
**3**	**Latency to Feed**				0.04
	**Aggression**				**5.62**
	**Shoaling Distance**				0.57
**4**	**Latency to Feed**				
	**Aggression**				
	**Shoaling Distance**				

Tank 1 (Flow, no vegetation), 2 (No Flow, vegetation), 3 (No flow, no vegetation) and 4 (Flow, Vegetation) for PN population. Significant differences (after bonferroni corrections for multiple comparisons) with p<0.01 are shown in bold.

**Table 5:** Paired comparisons of mean differences across tank treatment types (1, 2, 3 and 4) for (log transformed) Latency to feed, (square-root transformed) Aggression and (log transformed) Shoaling distance measurements.

Tank Type	Behaviour	1: Flow, No Veg	2: No Flow, Veg	3: No flow, No veg	4: Veg, Flow
**1**	**Latency to Feed**		0.20	1.10	0.52
	**Aggression**		2.33	**2.90**	3.57
	**Shoaling Distance**		0.42	**0.70**	0.22
**2**	**Latency to Feed**			1.30	0.70
	**Aggression**			**5.23**	1.24
	**Shoaling Distance**			0.28	0.20
**3**	**Latency to Feed**				0.59
	**Aggression**				**6.47**
	**Shoaling Distance**				0.48
**4**	**Latency to Feed**				
	**Aggression**				
	**Shoaling Distance**				

Tank 1 (Flow, no vegetation), 2 (No Flow, vegetation), 3 (No flow, no vegetation) and 4 (Flow, Vegetation) for SH population. Significant differences (after bonferroni corrections for multiple comparisons) with p<0.01 are shown in bold.

In contrast, Aggression was significantly different for all three populations when their respective responses were tested for effect of tank environments (Table 2). Post hoc (with Bonferroni adjustments for multiple comparisons, selection criterion α = 0.05/6) pairwise comparisons of responses showed significant differences (i.e., p<0.008) for several paired samples t- tests between tank types for all three populations (Tables 3, 4 and 5).

## Discussion

In environments that are unpredictable and variable, flexibility in behavior is critical [31]. The present study shows that zebrafish populations vary in their extent of behavioral plasticity, but that trends varied across types of behavior. Whereas Aggression varied significantly across environmental context for all three populations, Shoal Distance did not, suggesting that it is likely fixed by underlying physiological mechanism or developmental experience. Latency to Feed depended on population identity, with most of the variation being explained by domestication (the lab strain acclimated more quickly after being placed in a test arena). More interestingly, fish from the U stream population took different amounts of time to recover from disturbance depending on habitat, whereas fish from the PN lake population varied little in Latency to Feed across treatments.

While some behavior patterns may fluctuate readily with environment and social context, others are more likely to be determined by a hardwired mechanism (physiological and/ genetic pathways, early experience). Sih [32] indicated that learned and conditioned responses are typically dictated by early ‘rearing’ experiences and in such cases, an interaction of developmental and adult environment plays a major role in behavioral expression [33]. There are convincing arguments for plasticity as an agent of micro- and macroevolutionary change [34, 35, 36]. Plastic responses may be necessary for colonization of novel habitats [1, 35, 37]. For example, dietary plasticity is widespread and frequent in many land bird species during migration [38]. Being an adaptive advantage for a species to respond appropriately to new environments [39, 40], behavioral plasticity enhances their invasive abilities and adaptation to novel habitats.

Zebrafish prefer habitats that consist mostly of shallow, stagnant or slow moving waters [22]. They have been reported to occur in the Gangetic floodplain regions of north and eastern India. This region is subjected to seasonal rainfall regimes with a relatively dry winter and yearly monsoons during the summer. The wild populations used in this study have been collected from habitats that are a part of the streams and waterbodies of the Gangetic drainage system. During the monsoons (June- August) they are frequently subjected to sudden increase in water flow while the drier (November- May) months often result in near drying up of these habitats. There are corresponding changes to the vegetation in these habitats (higher vegetation during the post monsoon months) (pers. obs.). For survival in such seasonally changing environmental conditions, wild zebrafish would be expected to exhibit an ability to switch behavioural responses several times within a generation. In our study, zebrafish populations responded to changing contexts under laboratory conditions by exhibiting flexibility in aggression, but not so much for feeding and shoaling. Aggression increased with both vegetation and water flow. Earlier studies have indicated the role of habitat complexity (induced by vegetation) in reducing aggressive response (as complex habitats are more difficult to defend) in zebrafish [41]. A later study however showed that reduction in aggression and monopolization of resources could be a result of the extent of safety in these habitats and not just the effect of complexity and reduced defendability [42]. We found that although there were no significant differences between populations on overall aggression, both the wild and lab populations showed significant variability across treatments. We found lowest aggression among zebrafish in unvegetated still water tanks, for all three populations we tested. Unvegetated still water tanks might have been perceived as more risky, and therefore resulted in lower aggression levels. This result corresponds with [43] which found that adult aggression can change with social context. This flexibility in behavior across contexts suggests that there is an adaptive benefit of plasticity in aggression. In zebrafish populations, the ability to increase or decrease aggression levels according to the optimal requirement for a new environment can also help in their invasion of novel habitats [33].

We found that fish from the lab SH population fed more quickly after a disturbance in every tested treatment tank, confirming that this behavior is a good indicator of domestication, even when measured in groups of fish rather than individuals [44]. Both the wild populations (U and PN) showed significant differences from SH in each habitat type, while all comparisons between U and PN (except paired comparison in ‘still water-unvegetated’ tank) were not significant. This finding makes intuitive sense, since the lab populations are usually kept in generally resource rich environments and do not need to compete so hard with conspecifics for food. In comparison, wild populations typically occurring in resource deprived environments tend to feed more rapidly when food becomes available. Among the wild populations ‘U’ shoals exhibited significant variation in feeding latencies across treatments, while ‘PN’ and ‘SH’ shoals did not show significant variability.

Shoaling did not vary significantly across (habitat/environment) situations in any of the wild populations tested. Indeed, recent studies on zebrafish show that shoaling preference develops in juvenile zebrafish and once established, their preference remains stable across changing social environments [45]. SH fish do not shoal closely together, with larger distances between fish in the shoal than either of the two wild populations. This result is consistent with the greater ease with which SH fish left the vicinity of a shoal in comparison to two other strains measured in Moretz et al. [44]. Shoal distances, however did not vary significantly across tanks for any of the three populations. There are several advantages to shoaling and therefore these advantages can far override the disadvantages through competition for resources from conspecifics [46]. Indeed, Sih et al. [47] examined similar trends of correlated antipredatory behavior across situations among individuals of sunfish showing limited (or less optimal) behavioral plasticity. These apparently ‘non-adaptive phenomena’ have been argued as being maintained through behavioral correlations (across situations or contexts) that reflect underlying proximate mechanism (e.g. pleiotropic genes, common endocrine pathways, etc.) [32,48]. In our study, the consistent shoaling patterns observed across environmental contexts among zebrafish populations suggest a behavioral correlation for this trait with changes in flow and vegetation regimes.

To summarize, our findings indicate that expression of aggression is driven significantly by context or environmental factors, irrespective of population. On the other hand, latency to feed displayed significant dependence on population and not on environmental factors. Finally, shoaling distance was found to not depend on either population or environmental context. In this way, our experiments suggest differing levels of interactions between genetic (population level) and environmental factors determining behavioral responses. Indeed, recent findings suggest the importance of heritability in expression of aggression in zebrafish [49], as well as the interplay of genes and environment on such traits [50]. Thus, further experiments which study the effects of rearing conditions within genetically similar lines may shed more light on the role of physiological and genetic mechanisms in shaping these traits.

## Supporting Information

S1 File Latency to feed (in seconds) measurements for each population (U, PN and SH) across the four tank treatments (Tank1, Tank2, Tank3, Tank4). (XLS)

S2 File Aggression (number of chases) measurements for the populations (U, PN, SH) across the four tank treatments (Tank1, Tank2, Tank3, Tank4). (XLS)

S3 File Shoal Distance (in centimeters) measurements for the populations (U, PN, SH) across the four tank treatments (Tank1, Tank2, Tank3, Tank4). (XLS)
